# Targeting the chromatin remodeling enzyme BRG1 increases the efficacy of chemotherapy drugs in breast cancer cells

**DOI:** 10.18632/oncotarget.8384

**Published:** 2016-03-25

**Authors:** Qiong Wu, Soni Sharma, Hang Cui, Scott E. LeBlanc, Hong Zhang, Rohini Muthuswami, Jeffrey A. Nickerson, Anthony N. Imbalzano

**Affiliations:** ^1^ Department of Cell and Developmental Biology, University of Massachusetts Medical School, Worcester, MA, USA; ^2^ School of Life Sciences, Jawaharlal Nehru University, New Delhi, Delhi, India; ^3^ Abace Biotech Co Ltd., Yi Zhuang Biomedical Park, BDA, Beijing, China

**Keywords:** epigenetics, BRG1, SWI/SNF, breast cancer, drug transporters

## Abstract

Brahma related gene product 1 (BRG1) is an ATPase that drives the catalytic activity of a subset of the mammalian SWI/SNF chromatin remodeling enzymes. BRG1 is overexpressed in most human breast cancer tumors without evidence of mutation and is required for breast cancer cell proliferation. We demonstrate that knockdown of BRG1 sensitized triple negative breast cancer cells to chemotherapeutic drugs used to treat breast cancer. An inhibitor of the BRG1 bromodomain had no effect on breast cancer cell viability, but an inhibitory molecule that targets the BRG1 ATPase activity recapitulated the increased drug efficacy observed in the presence of BRG1 knockdown. We further demonstrate that inhibition of BRG1 ATPase activity blocks the induction of ABC transporter genes by these chemotherapeutic drugs and that BRG1 binds to ABC transporter gene promoters. This inhibition increased intracellular concentrations of the drugs, providing a likely mechanism for the increased chemosensitivity. Since ABC transporters and their induction by chemotherapy drugs are a major cause of chemoresistance and treatment failure, these results support the idea that targeting the enzymatic activity of BRG1 would be an effective adjuvant therapy for breast cancer.

## INTRODUCTION

Drugs that target genomically defined vulnerabilities in human tumors have been effective cancer therapies for decades [1]. The specificity of cancer drugs has progressively improved, from general cytotoxic agents such as nitrogen mustard in the 1940s [2] to natural-product anticancer drugs such as *Vinca* alkaloids and anthracyclines in the 1960s [3], to specific monoclonal antibodies [4], immunotoxins [5], and small molecules targeting cell surface receptors and growth-promoting signal transduction pathways [6]. Increased specificity has improved patient response rates while reducing the side effects of anticancer treatment. However, the rapid acquisition of resistance to drug treatments remains a substantial challenge to the clinical management of advanced cancers. Resistance to single drugs can be overcome by combinatorial treatment with drugs acting *via* different mechanisms, but cancer cells often evolve simultaneous resistance to different structurally and functionally unrelated drugs, a phenomenon known as multidrug resistance (MDR) [7, 8]. Resistance to anticancer drugs arises by various mechanisms and especially by the genetic instability of tumor cells driving heterogeneity. While therapies have become more targeted and effective, acquired resistance has remained the principal basis for treatment failure [9, 10].

One common reason for resistance to multiple anticancer drugs is the increased expression of one or more energy-dependent transporters that result in efflux of the drugs from cells [11, 12]. The first identification of a molecular mechanism of multidrug resistance was the identification of an energy-dependent drug efflux pump, known as P-glycoprotein (P-gp) or MDR1, the multidrug transporter [13, 14]. The product of the human MDR1 gene [15] and the products of two different but related mouse genes, Mdr1a and Mdr1b [16, 17], were among the first described members of a large family of ATP-dependent transporters known as the ATP-binding cassette (ABC) family [18]. From the 48 known ABC transporters [19], members of three subfamilies are important for drug efflux from cells: (i) MDR1 P-glycoprotein (ABCB1) from the “B” subfamily, which was the first identified ABC drug efflux transporter and has been the most completely characterized [11]; (ii) several multidrug resistance related protein (MRP) transporters from the “C” subfamily (ABCC1, ABCC2, ABCC3, ABCC4, ABCC5, ABCC10, ABCC11) [20–22] and (iii) ABCG2/BCRP from the “G” subfamily [23].

The SWI/SNF enzymes control gene expression through ATP-dependent remodeling of chromatin. Mammalian SWI/SNF complexes contain mutually exclusive ATPase subunits, either BRM (SMARCA2), or BRG1 (SMARCA4) [24–26]. SWI/SNF complexes containing BRG1 control cell proliferation, cell lineage differentiation and maintain cell pluripotency during early embryonic development [27–33]. A growing body of evidence suggests that BRG1 exhibits both tumor suppressing and tumor promoting functions, depending on the type of cancer [32]. Results published by us and by others demonstrate that the SWI/SNF ATPases BRG1 and BRM are up-regulated in primary breast cancer and are required for cancer cell proliferation *in vitro* and *in vivo* [27, 33]. These results suggest that BRG1, as a driver of proliferation, could be a drugable target in certain cancer types. In addition, BRG1 promotes chemoresistance in lung cancer cells [34], where BRG1 wildtype tumors upregulate BRG1 in response to EZH2 inhibitor and become more resistant to TOPOII inhibitor. In pancreatic tumors, BRG1 knockdown effectively reverses chemoresistance to gemcitabine [35].

Breast cancer is the most common cancer in women and one of the leading causes of cancer death for women, with triple negative breast cancer being the most invasive and life threatening [36–39]. Triple negative breast cancer has been shown to be highly glycolytic, metastatic, and chemotherapy resistant; currently there are no standard of care effective targeted therapies to combat triple negative breast cancer. Therefore, both early stage and advanced triple negative breast cancer tumors are treated with predominantly cytotoxic chemotherapy. We previously reported that reduction of BRG1 results in slow proliferation in triple negative breast cancer cells *in vitro* and in xenografts [33]. We report here that depletion of BRG1 or an inhibitor targeting the BRG1 ATPase domain sensitized triple negative breast cancer cells to chemotherapeutic drugs. BRG1 inhibition prevented chemotherapy drug-mediated induction of genes encoding specific ABC transporter proteins. We conclude that targeting the ATPase domain of BRG1, in combination with other chemotherapy drugs, is a promising strategy for breast cancer treatment.

## RESULTS

Breast tumors are heterogeneous with subtypes defined by pathology [40] and gene expression profiles [41]. Since we were studying chemotherapy drug efflux,we chose to focus on the subtype with the most resistance to those drugs, the most treatment failures, and the worst prognosis for patients [36]. These ‘triple negative' tumors lack estrogen receptor α, lack the progesterone receptor, and do not have HER2 upregulation.

### BRG1 depletion sensitized triple negative breast cancer cells to chemotherapy drugs

**Figure 1:**
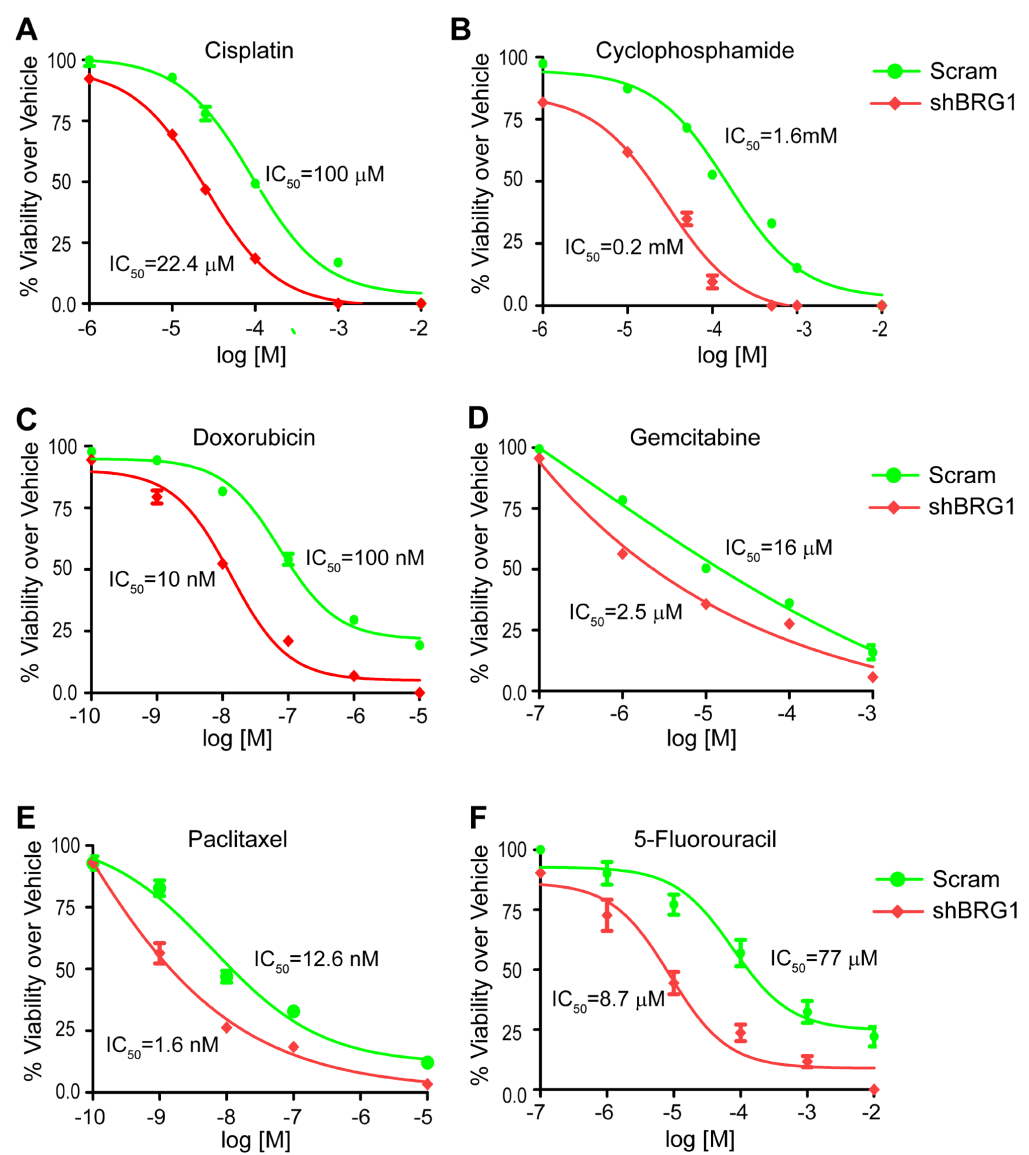
Reduction of BRG1 levels increased the chemosensitivity of triple-negative breast cancer cells BRG1 knockdown was induced by Doxycycline in MDA-MB-231 cells engineered as previously described [33]. Cells were plated in 96-well plates with increasing doses of cisplatin **A.**, cyclophosphamide **B.**, doxorubicin **C.**, gemcitabine **D.**, paclitaxel **E.**, or 5-fluorouracil **F.** for 72 hours. Cell viability was measured by MTS assay. Fold change over vehicle treated cells was calculated. IC50 values were determined by nonlinear regression analysis using a log (inhibitor) *vs* response (three parameters) model in GraphPad Prism 6.0. The results are presented as the mean of three independent experiments performed in triplicate. Error bars are standard deviations.

We tested the efficacy of six chemotherapy drugs on MDA-MB-231 cells expressing doxycyline-inducible shRNA targeting BRG1. Doxorubicin, 5-fluorouracil (5-FU), gemcitabine, cisplatin, cyclophosphamide, and paclitaxel are used clinically for treating breast cancer patients. Doxorubicin induces DNA double-strand breaks. 5-FU and gemcitabine are nucleoside analogs. Cisplatin crosslinks DNA, while cyclophosphamide alkylates and crosslinks DNA. Paclitaxel prevents normal breakdown of microtubules during mitosis. These drugs are structurally unrelated and have different mechanisms of action. Reduction of BRG1 expression significantly improved the efficacy of each of these drugs (Figure 1A-1F, Supplemental Figure 1A) as judged by cell viability in MTS assays [42]. The IC50 values were decreased 4 to 10 fold, supporting the concept that BRG1 reduction or inhibition might be effective as an adjunct to currently used chemotherapies.

### A BRG1 ATPase domain inhibitor decreased breast cancer cell proliferation

**Figure 2:**
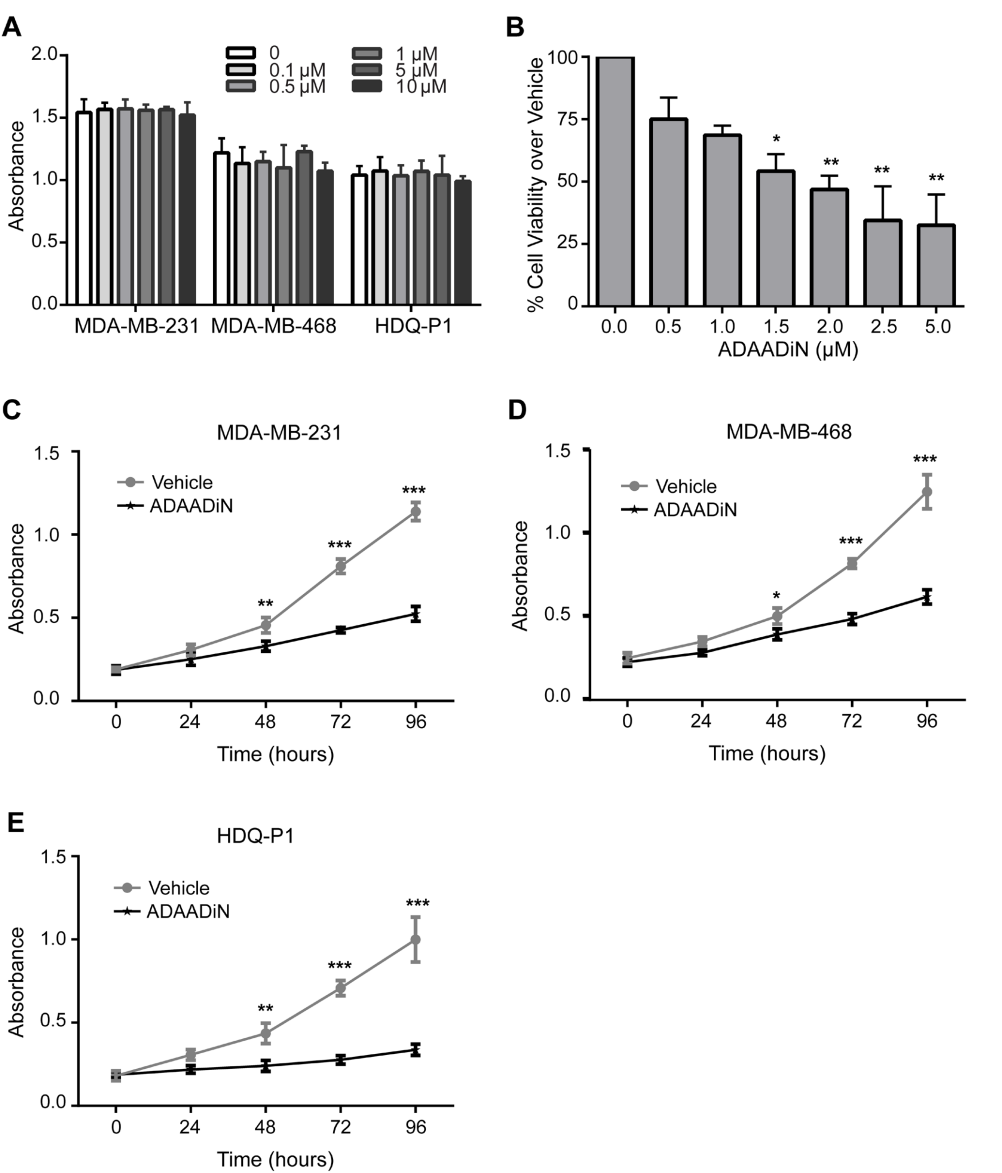
Effects of BRG1 inhibitors on breast cancer cell proliferation and viability **A.** Cell proliferation was measured by MTT assay in three triple negative breast cancer cell lines (MDA-MB-231, MDA-MB-468 and HDQ-P1) with increasing doses of PFI-3 treatment for 72 hours. **B.** Cell viability in MDA-MB-231 cells treated with increasing doses of ADAADi was examined by MTT assay for 48 hours. MTT assays were performed on MDA-MB-231 **C.**, MDA-MB-468 **D.** and HDQ-P1 **E.** cells treated with 2 μM ADAADi. Each data point represents the mean of 3 independent experiments performed in triplicate. Error bars are standard deviations. *P < 0.05, **P < 0.01, ***P < 0.001.

Only two BRG1 inhibitors have been reported. PFI-3, or (2E)-1-(2-hydroxyphenyl)-3-[(1R,4R)-5-(pyridin-2-yl)-2,5-diazabicyclo[2.2.1]heptan-2-yl]prop-2-en-1-one, a small molecule inhibitor that specifically targets the bromo domains of BRG1, BRM, and PB1, is a Pfizer/ Structural Genomics Consortium candidate with *in vitro* potency in isothermal titration calorimetry at < 100 nM dose (http://www.thesgc.org/chemical-probes/PFI-3; [43, 44]). Previously, we reported that depletion of BRG1 in triple negative breast cancer cell lines reduced cell proliferation [33]. We treated three triple negative breast cancer cell lines, MDA-MB-231, MDA-MB-468, and HDQ-P1, with PFI-3 at different doses. No inhibition of cell proliferation was observed, even at the 10 μM dose as measured by MTT assay [45] (Figure 2A). This is consistent with recent results demonstrating that PFI-3 did not affect the proliferation rate of other cancer cell lines [44].

**Figure 3:**
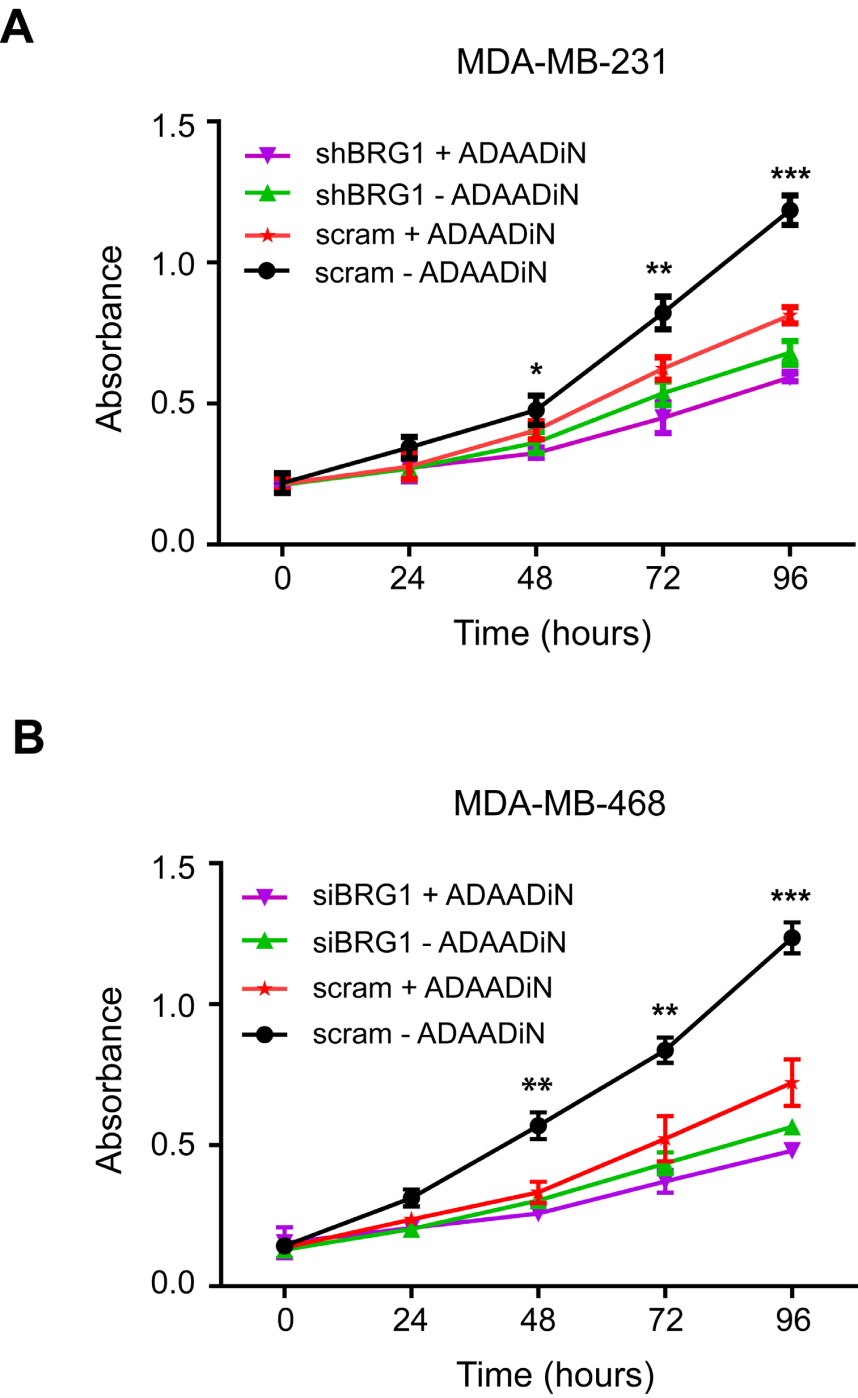
ADAADiN-mediated inhibition of triple negative breast cancer cell proliferation and viability is due to inhibition of BRG1 **A.** Cell proliferation was measured by MTT assay after ADAADi treatment, inducible BRG1 knockdown, or both in MDA-MB-231 cells. **B**. Cell proliferation was measured by MTT assay after ADAADi treatment, BRG1 knockdown by siRNA, or both in MDA-MB-468 cells. Each data point is the mean from 3 independent experiments performed in triplicate; error bars are standard deviations. *P < 0.05, **P < 0.01, ***P < 0.001.

The bacterial APH (3′)-III enzyme that encodes for aminoglycoside resistance generates a minor product that can be chromatographically separated from the known 3′-phosphoaminoglycoside product as well as from the parental aminoglycoside. This product, named ADAADi (Active DNA-dependent ATPase A Domain inhibitor), inhibits the ATPase activity of the SWI2/SNF2 family of ATPases [46, 47]. Enzymes from other families of DNA-dependent ATPases showed no or greatly reduced sensitivity to ADAADi, and DNA-independent or RNA-dependent ATPases were not affected [47]. Prior studies indicated that ADAADi inhibited BRG1 nucleosome remodeling activity *in vitro* [47]. ADAADi derived from different aminoglycosides behaves similarly in all tested assays [46]; here we utilized ADAADi derived from neomycin (ADAADiN). We tested the ADAADiN inhibitor on three triple negative lines: MDA-MB-231, MDA-MB-468 and HDQ-P1. ADAADiN significantly decreased cell proliferation in these cell lines (Figure 2B-2E). However, ADAADiN failed to decrease cell proliferation significantly in cells with reduced BRG1 expression (Figure 3A-3B; Supplemental Figure 1B). This observation strongly suggests that ADAADiN targeted BRG1 in these cells by interfering with its ATPase function.

Brahma (BRM) is highly homologous to BRG1 [25, 48] and can also function as the catalytic subunit of mammalian SWI/SNF enzymes in a manner mutually exclusive of BRG1 [26]. ADAADiN decreased cell proliferation to roughly the same extent as shRNA mediated knockdown of BRM (Supplemental Figure 1 and 2). However, the combination of ADAADiN and shRNA targeting BRM further decreased proliferation in a manner that is statistically significant and additive (Supplemental Figure 2). This finding is in contrast to the results obtained for treatment of cells with a combination of ADAADiN and shRNA targeting BRG1 (Figure 3) and suggests that ADAADiN specifically targets BRG1 in these cells.

### ADAADiN treatment increased breast cancer cell sensitivity to chemotherapeutic drugs

Since ADAADiN inhibited breast cancer cell proliferation, we asked if it could also sensitize cells to chemotherapeutic drugs, just as BRG1 knockdown does. Following pretreatment with ADAADiN, cells were exposed to different doses of the same chemotherapy drugs, and cell viability was assayed by MTT assay. ADAADiN significantly increased the chemotherapeutic sensitivity of MDA-MB-231 and MDA-MB-468 cells from 3- to well over 10-fold (Table 1). These data establish the concept that chemical inhibition of the BRG1 ATPase domain might be used to target BRG1 mediated pro-survival pathways in breast cancer cells.

**Table 1:** ADAADiN significantly increased the sensitivity of MDA-MB-231 and MDA-MB-468 to chemotherapy drugs

	MDA-MB-231		MDA-MB-468	
	Vehicle	ADAADi	Vehicle	ADAADi
5-fluorouracil	16.9 ± 4.6 μM	4.2 ± 1.1 μM	27.15 ± 5.45 μM	5.22 ± 0.63 μM
Cisplatin	5.4 ± 0.8 μM	0.14 ± 0.03 μM	1.94 ± 0.27 μM	0.37 ± 0.08 μM
Cyclophosphamide	1.45 ± 0.15 mM	0.061 ± 0.005 mM	0.4 ± 0.02 mM	0.028 ± 0.003 mM
Doxorubicin	68.13 ± 5.11 nM	7.16 ± 1.23 nM	60.3 ± 4.9 nM	8.12 ± 3.7 nM
Gemcitabine	166 ± 10.8 nM	59.9 ± 7.2 nM	2.21 ± 0.33 μM	0.42 ± 0.015 μM
Paclitaxel	29.25 ± 7.49 nM	1.35 ± 0.52 nM	20.55 ± 1.95 nM	1.21 ± 0.19 nM

Shown are IC_50_ doses for each drug and cell line.

### ADAADiN blocked induction of drug transporter gene expression in response to drug treatment

ABC transporters mediate the efflux of anti-cancer drugs and are critically involved in multidrug resistance [21, 49–51]; the expression of ABC transporters is up-regulated in patients after neoadjuvant therapy [52]. We first surveyed nine ABC transporter genes to determine whether BRG1 contributed to their expression in MDA-MB-231 cells. The results show that BRG1 contributed to the endogenous level of transporter gene expression for seven of the genes (Supplemental Figure 3).

Since ADAADiN sensitized breast cancer cells to chemotherapeutic drugs, we hypothesized that ADAADiN treatment might inhibit the transcriptional activation of the transporter genes upon chemotherapy drug treatment. From the literature, we identified six instances where ABC transporter genes are transcriptionally activated in response to one or more of the chemotherapeutic drugs used in our study. Each of the triple negative breast cancer cell lines were treated with vehicle alone or with one of the chemotherapy drugs at the IC50, dose and specific transporter mRNA levels were compared to levels present in cells exposed to drug plus ADAADiN.

**Figure 4:**
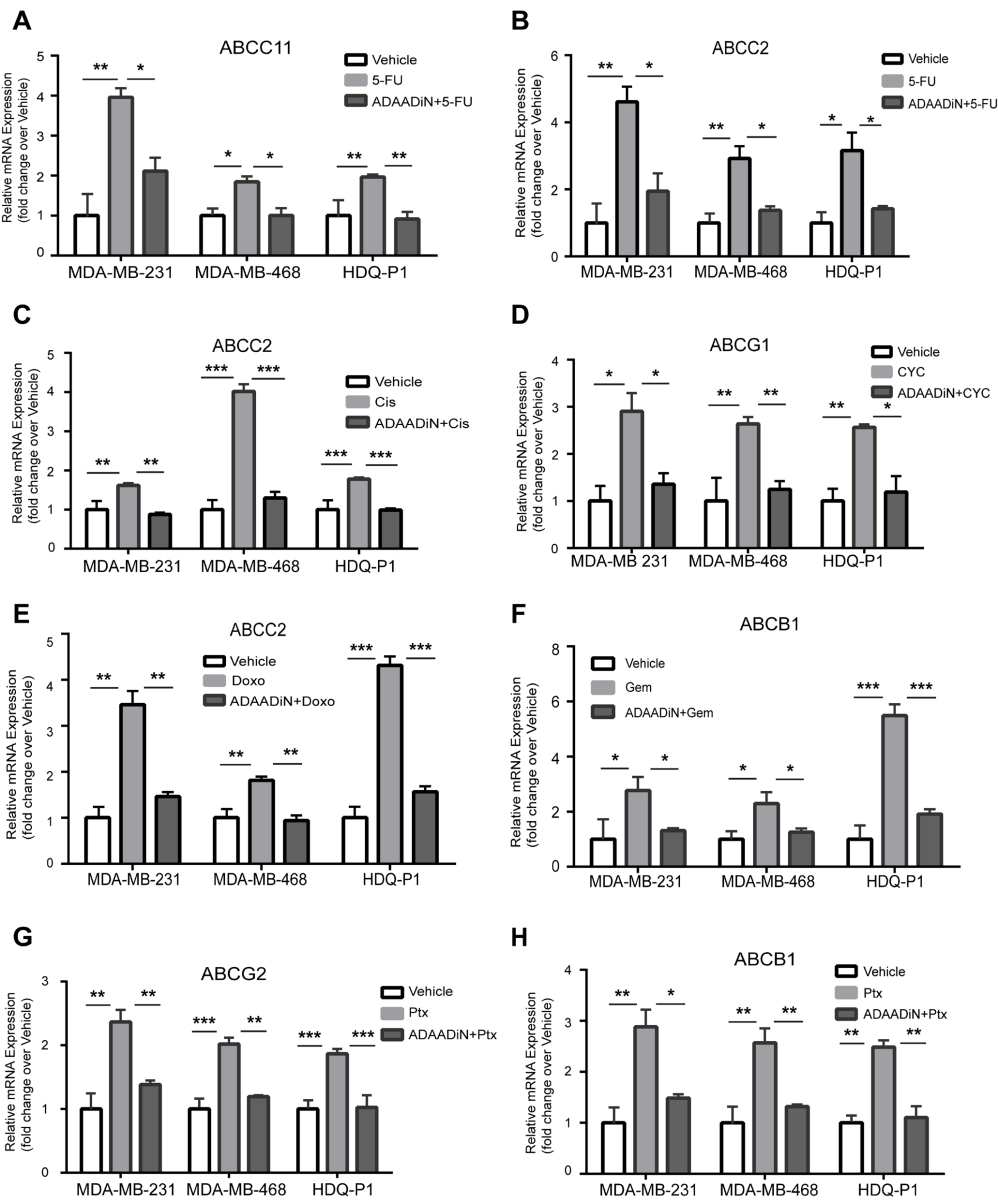
ADAADiN blocked drug-induced ABC transporter gene expression in triple negative breast cancer cell lines Bar graphs present relative mRNA expression of specific ABC transporter genes in response to vehicle, the indicated chemotherapeutic drug, and the indicated chemotherapeutic drug in the presence of ADAADiN in each of the indicated breast cancer cell lines. Each bar presents the mean of 3 independent experiments performed in duplicate; error bars are standard deviations. *P < 0.05, **P < 0.01, ***P < 0.001. **A.**-**B.** 5-FU mediated induction of ABCC11 and ABCC2. **C.** Cisplatin (Cis) mediated induction of ABCC2. **D.** Cyclophosphamide (CYC) mediated induction of ABCG1. **E.** Doxorubicin (Doxo) mediated induction of ABCC2. **F.** Gemcitabine (Gem) mediated induction of ABCB1. **G.**-**H.** Paclitaxel (Ptx) mediated induction of ABCG2 and ABCB1.

ABCC11 was previously identified as a 5-FU efflux transporter that directly confers resistance to 5-FU [53, 54]. We observed up-regulation of ABCC11 expression in each of the three triple negative cell lines when treated with 5-FU, and this activation was attenuated in the presence of ADAADiN (Figure 4A).

Minegaki et al [55] reported that ABCC2 mRNA levels increased in a dose-dependent manner when treated with 5-FU. ABCC2 expression was increased more than 2-fold in all three cell lines when treated with an IC50 dose of 5-FU. When co-treated with ADAADiN, ABCC2 mRNA levels were significantly decreased (Figure 4B). ABCC2 also mediates cisplatin resistance and this is correlated with clinical outcome [56, 57]. In our study, cisplatin up-regulated ABCC2 expression by 4-fold in MDA-MB-468 cells. Activation in MDA-MB-231 and HDQ-P1 cells was less than 2-fold but was nevertheless statistically significant. However, the up-regulation of ABCC2 in each of the three cell lines was inhibited by co-treatment with ADAADiN (Figure 4C).

The expression of ABCG1 is increased in breast cancer patients during neoadjuvant therapy with 5-fluorouracil-doxorubicin-cyclophosphamide, and increased levels of ABCG1 predict poor prognosis [58]. ABCG1 expression increased in each of the cell lines when treated with cyclophosphamide, while the presence of ADAADiN suppressed ABCG1 up-regulation (Figure 4D).

Targeting ABCC2 by antisense RNA reduces the doxorubicin IC50 value by 12-fold in hepatocellular carcinoma cells, whereas overexpression of ABCC2 in HEK293 cells enhances doxorubicin resistance around 8-fold [56, 59]. These results suggest that ABCC2 is the efflux transporter for doxorubicin. When the three triple negative breast cancer lines were treated with the IC50 dose of doxorubicin, it caused a greater than 3-fold induction of ABCC2 in MDA-MB-231 and HDQ-P1 cells, and a modest but statistically significant increase in MDA-MB-468 cells. The addition of ADAADiN blocked ABCC2 induction in each cell line (Figure 4E).

ABCB1 is overexpressed in gemcitabine resistant pancreatic cells and in side population cells with high gemcitabine efflux capacity [60, 61]. In non-small-cell lung cancer cells, ABCB1 mRNA levels can predict gemcitabine chemosensitivity [62]. Homology modeling and docking of ABCB1 showed gemcitabine to be a high-affinity substrate [63]. In the three triple negative lines tested, ABCB1 was strongly induced by gemcitabine treatment, but ADAADiN treatment effectively blocked its induction (Figure 4F).

EGFR-mediated overexpression of ABCG2 is associated with paclitaxel resistance in drug resistant MCF-7 cells [64]. Silencing ABCB1 and ABCG2 by nanoparticle-facilitated siRNA in MCF-7 cells increases chemosensitivity to paclitaxel [65, 66]. As shown in Figure 4G-4H, the expression of ABCB1 and ABCG2 was up-regulated in each of the three cell lines treated with paclitaxel. However, the presence of ADAADiN significantly blocked the activation of these genes. In summary, ADAADiN treatment blocked the transcriptional induction of drug transporters by chemotherapeutic drugs, which may contribute to the increase chemosensivity in ADAADiN treated cells.

### Targeting BRG1 led to an increase in drug retention

**Figure 5:**
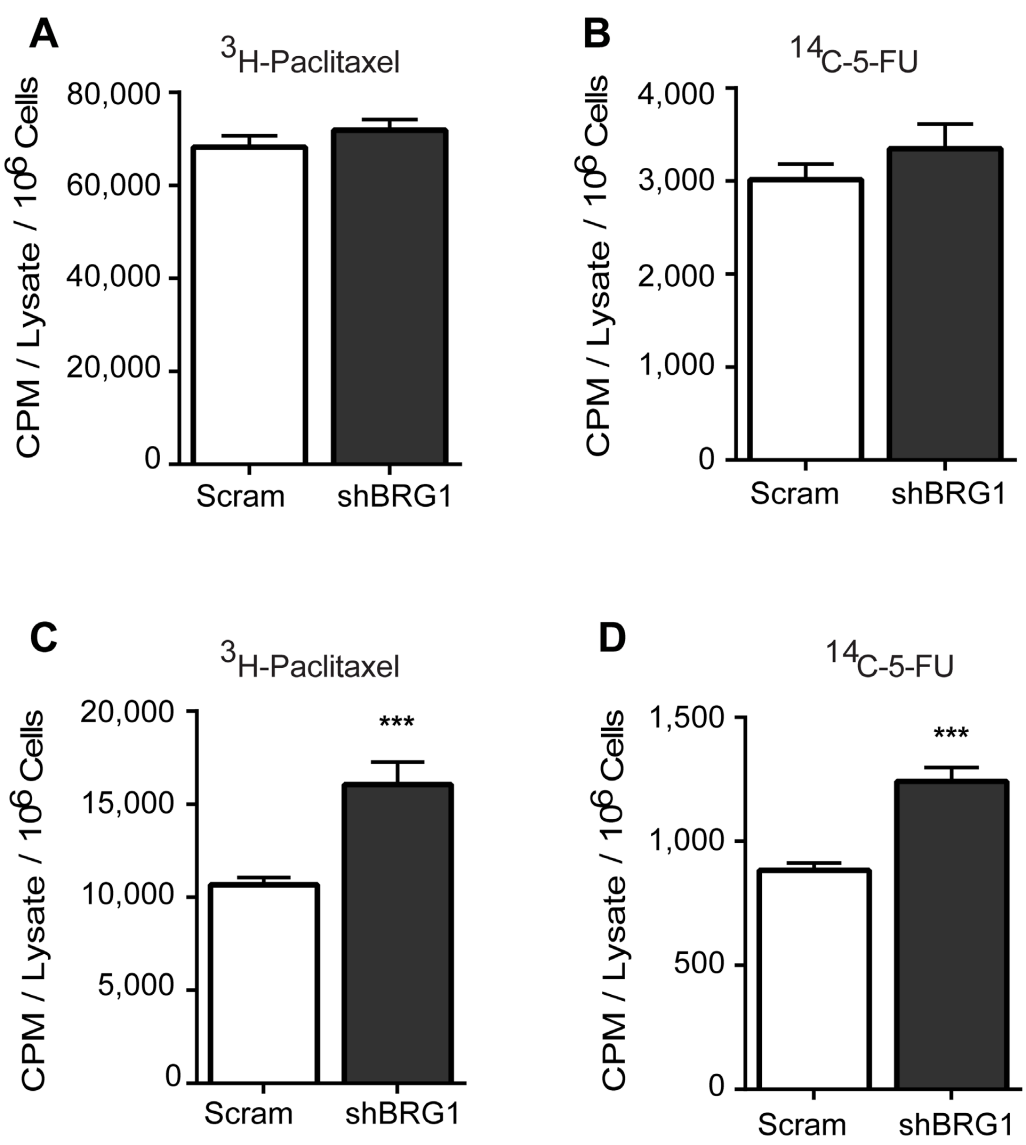
Targeting BRG1 results in increased retention of chemotherapeutic drugs Radiolabeled paclitaxel or 5-FU were incubated with MDA-MB-231 cells expressing a control (Scram) shRNA or shRNA targeting BRG1. **A.**-**B.** Cellular uptake was determined by scintillation counting after harvested cells were washed repeatedly, counted, and lysed. Results were normalized by cell count. **C.**-**D.** Drug retention was determined by scintillation counting after cells were pulse-chased and prepared as described in Materials and Methods. Each bar presents the mean of 3 independent experiments performed in triplicate; error bars are standard deviations. ***P < 0.001.

Altering drug efflux transporter expression might change the intracellular concentration of the chemotherapeutic drugs. Because of the limited availability of radiolabeled versions of the drugs used in this study, we used only ^14^C-5-FU and ^3^H-paclitaxel to test this hypothesis. The uptake of the radiolabeled drugs was identical in cell cultures expressing control shRNA or shRNA targeting BRG1 (Figure 5A-5B). Subsequent analysis of cell cultures that were pulse-chased with the drugs showed that BRG1 knockdown resulted in increased intracellular retention of the drugs (Figure 5C-5D). We conclude that BRG1-dependent induction of drug transporter gene expression by 5-FU and Paclitaxel results in increased intracellular concentrations of the drugs. This may explain the increased chemosensitivity of cells that express reduced levels of BRG1 or that are treated with a BRG1 inhibitor.

### Drug treatment increased BRG1 binding at drug transporter promoters

**Figure 6:**
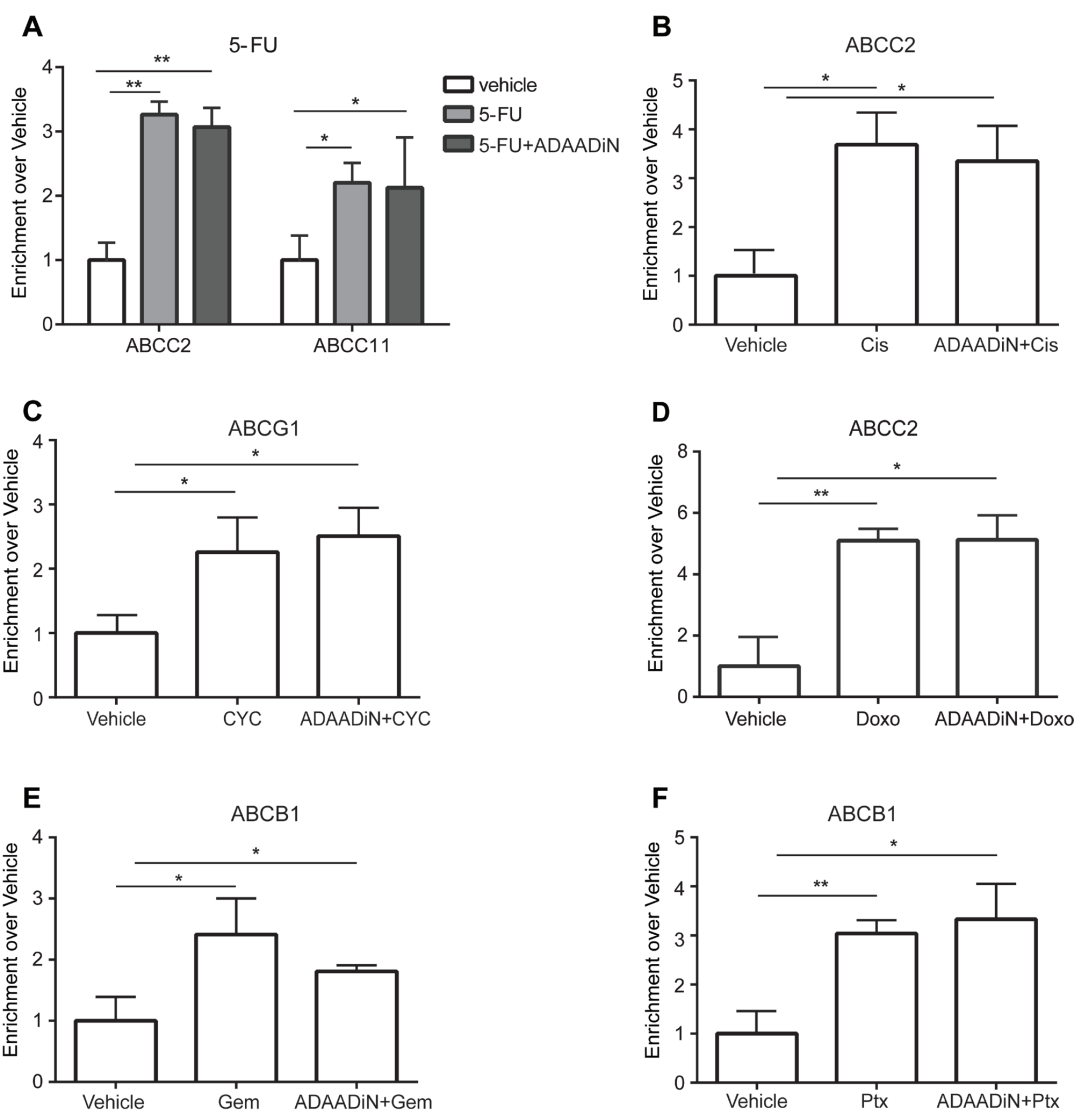
Chemotherapeutic drugs increased BRG1 binding at ABC transporter genes in a manner independent of ADAADiN ChIP was performed in MDA-MB-231 cells treated with vehicle, chemotherapy drug alone or chemotherapy drug in combination with ADAADi. BRG1 binding at transporter genes was measured by quantitative PCR using primers listed in Supplemental Table 2. The bar graphs represent the ratio (enrichment) of BRG1 binding to sequences near the indicated transporter gene in cells treated with the indicated chemotherapeutic drug alone or in combination with ADAADi relative to binding in vehicle treated cells. Each bar presents the mean of 3 independent experiments performed in duplicate; error bars are standard deviations. *P < 0.05, **P < 0.01, ***P < 0.001. **A.** BRG1 binding at the ABCC2 and ABCC11 genes was analyzed in vehicle, 5-FU and ADAADi plus 5-FU treated cells. **B.** BRG1 binding at ABCC2 was analyzed in vehicle, cisplatin (Cis) and ADAADi plus Cis treated cells. **C.** BRG1 binding at ABCG1 was analyzed in vehicle, cyclophosphamide (CYC) and ADAADi plus CYC treated cells. **D.** BRG1 binding at ABCC2 was analyzed in vehicle, Doxorubicin (Doxo) and ADAADi plus Doxo treated cells. **E.** BRG1 binding at ABCB1 was analyzed in vehicle, gemcitabine (Gem) and ADAADi plus Gem treated cells. **F.** BRG1 binding at ABCB1 was analyzed in vehicle, paclitaxel (Ptx) and ADAADi plus Ptx treated cells.

SWI/SNF enzymes regulate gene expression by altering chromatin structure, and BRG1 binds to chromatin at many genes that are actively transcribed [30, 32]. We asked whether BRG1 is directly involved in the drug-induced transcriptional activation of the tested transporters. Binding of BRG1 at transporter genes was examined by chromatin immunoprecipitation (ChIP) in MDA-MB-231 cells treated with vehicle or with individual chemotherapeutic drugs at the IC50 dose, and these results were compared to results from cells treated with ADAADiN prior to and during induction with the chemotherapeutic drug. BRG1 binding sites at transporter genes promoters were predicted from BRG1 ChIP-seq data deposited for HeLa and K562 cells [67, 68]. 5-FU treatment enhanced BRG1 binding at ABCC2 by 3-fold and ABCC11 by 2-fold, and ADAADiN had no effect on BRG1 binding at these genes (Figure 6A). This result is consistent with the idea that ADAADiN inhibits ATPase activity but has no effect on the ability of the enzyme to bind to chromatin [46]. Cisplatin increased BRG1 enrichment at ABCC2 more than 3-fold, and co-treatment with ADAADiN did not change BRG1 binding (Figure 6B). Similar results were seen in cyclophosphamide, doxorubicine, gemcitabine and paclitaxel treated cells, where these drugs significantly increased BRG1 binding at target drug transporter genes and ADAADiN showed no effect on BRG1 binding (Figure 6C-6F). We were unable to examine BRG1 binding at the ABCG2 locus in paclitaxel treated cells, as there were no BRG1 binding sites identified at this locus in reported ChIP-seq data sets in different cell contexts. Overall, chemotherapeutic drugs increased BRG1 binding to drug transporter genes, and these binding events were not affected by the ADAADiN BRG1 ATPase inhibitor.

PFI-3, a compound targeting the bromodomain of BRG1, BRM, and PB1 [44] had no effect on proliferation of any of the breast cancer cell lines tested (Figure 2A). This is consistent with recent work of others using different cancer cell lines [44] showing that PFI-3 was unable to dislodge BRM from chromatin, either globally or at specific gene loci. We performed ChIP assays to address whether BRG1 remained bound in the presence of PFI-3. Eight transporter genes were examined for the binding of BRG1 in proliferating MDA-MB-231 cells. Four of the eight transporter genes tested had BRG1 binding. PFI-3 had no effect on the extent of BRG1 binding observed at any of the loci (Supplemental Figure 4). These data are consistent with the prior observation that PFI-3 could not displace BRM from chromatin [44] and extend those findings by showing that PFI-3 also cannot dislodge BRG1 from chromatin.

### BRG1 expression level correlated with breast cancer patient survival

**Figure 7:**
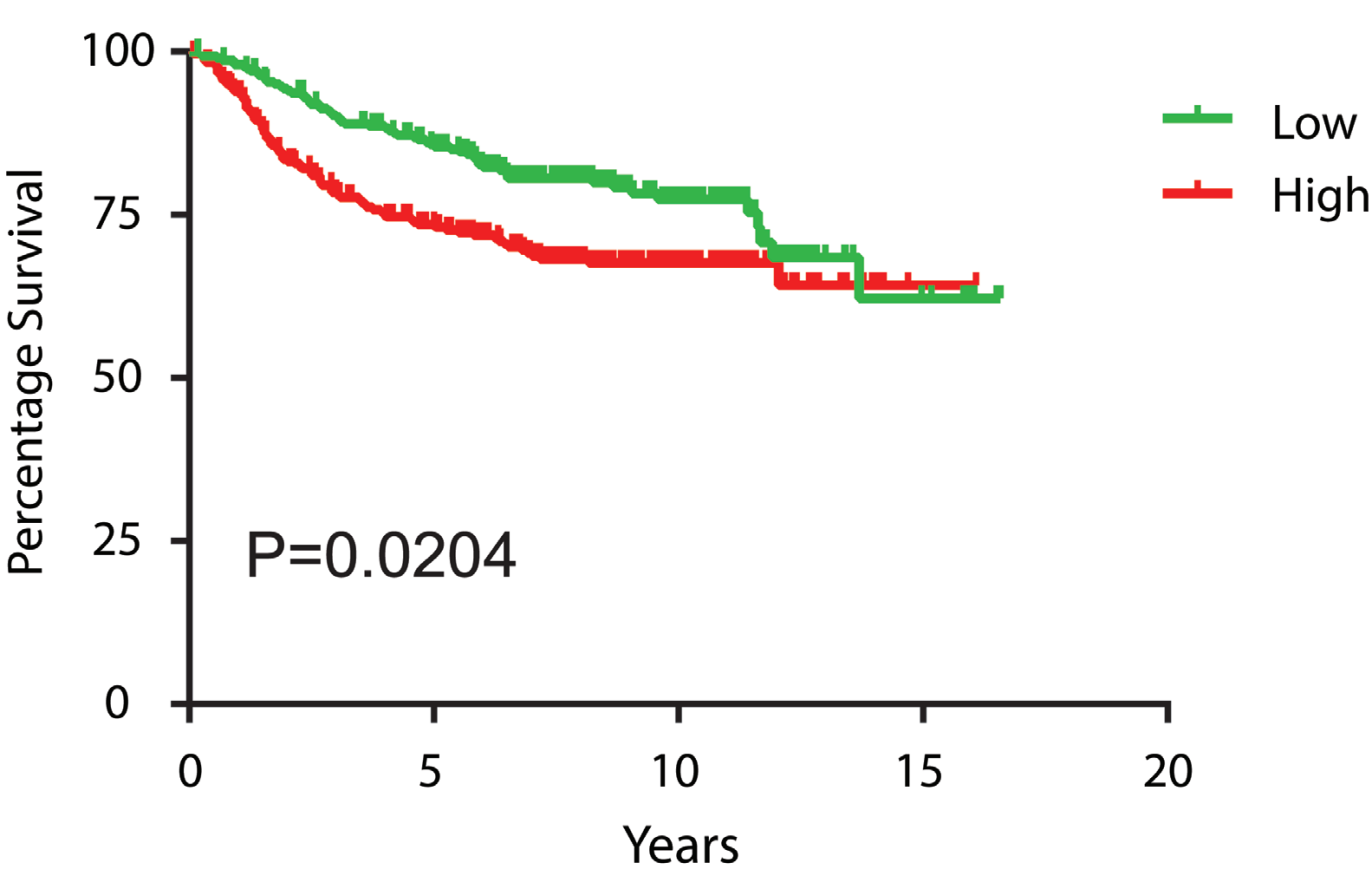
High BRG1 expression levels in breast tumors predicts poor patient prognosis Kaplan-Meier curves of distance metastasis free survival of human breast cancer patients from 7 combined datasets were plotted according to the level of BRG1 expression, with the 1st quartile having the lowest BRG1 expression designated as “low” and 4^th^ quartile having the highest BRG1 expression designated as “high”. The log-rank test was used for statistical analysis.

To determine the correlation between BRG1 expression and breast cancer patient survival, we retrieved 7 microarray datasets of human breast cancer (GSE1456, GSE2034, GSE2990, GSE3494, GSE12093, GSE11121 and Chin et al., combined *N* = 1339) profiled on Affymetrix HG-U133A platform [69–75] from Gene Expression Omnibus (GEO) (http://www.ncbi.nlm.nih.gov/geo) and European Bioinformatics Institute (http://www.ebi.ac.uk/arrayexpress for Chin et al, 2006 [75]). We normalized these datasets using MAS 5.0 software in GeneSpring 12.0 (Agilent Technologies, Santa Clara, CA), scaled to a mean target intensity of 600, and log_2_ transformed. 88 samples from GSE2990 were also used in GSE3494, and thus excluded from GSE2990. 23 samples without survival information and 7 samples with distance metastasis-free survival of 0 were excluded, which left 1221 non-redundant samples for survival analysis. There was a negative correlation between BRG1 expression level and patient prognosis, in which high levels of BRG1 were associated with poor prognosis (Figure 7). This result provides independent corroboration of a prior study [27] linking BRG1 levels with poor clinical outcomes of breast cancer patients. That conclusion, like our analysis of patient data here, was not limited to triple negative tumors, which make up only about 15% of patient tumors. Future studies will need to determine whether specific breast tumor subtypes have a negative BRG1 level correlation with prognosis.

Our results show that BRG1 knockdown or inhibition increases chemosensitivity and decreases drug-induced increases in ABC transporter gene expression. This suggests that BRG1 overexpression that is normally observed in primary breast tumors [27, 33] may result in elevated ABC transporter gene expression and possible chemoresistance. We might therefore expect that transporter gene expression would correlate with BRG1 expression. The microarray datasets used for correlating high BRG1 expression with decreased survival were interrogated for expression levels of the transporter genes that were stimulated by chemotherapeutic drugs in a BRG1-dependent manner (Figure 4). Three of these genes (ABCB1, ABCC2, ABCG2) showed increased levels of expression that correlated with BRG1 expression, whereas one of the genes (ABCG1) showed an inverse correlation (Supplemental Figure 5). The fifth gene, ABCC11, was not present in the microarray datasets. It is important to remember, however, that levels of specific transporter gene expression were generated from the combined dataset representing patients with a spectrum of breast cancers and that the majority of samples were isolated upon biopsy prior to any treatment. Nevertheless, this analysis provides support for the idea that ABC transporter gene expression is linked to BRG1 expression.

## DISCUSSION

### Small molecule inhibition of the BRG1 ATPase domain is a promising therapeutic strategy

Previous work by us and others indicates that BRG1 is overexpressed in most breast tumors regardless of classification and that BRG1 knockdown in triple negative breast cancer cells caused a slow proliferation phenotype [27, 33]. Here we report that BRG1 knockdown cells have increased sensitivity to chemotherapeutic drugs currently used to treat breast cancer (Figure 1), suggesting that targeting BRG1 may be a viable approach to augmenting current therapeutic regimens.

Delivery to and continued expression of knockdown vectors in most tissues and specifically in tumors presents significant challenges [76]. Identification of small molecule inhibitors, in contrast, has been an effective therapeutic approach for decades. PFI-3 is a cell-permeable small molecule inhibitor that specifically targets the bromodomains of BRG1, BRM, and a third mammalian SWI/SNF subunit, Polybromo (BAF180) *via* tight interaction [44, 77]. Bromodomains bind to acetylated chromatin and therefore have a targetable molecular function [78–80]. A recent study showed that PFI-3 treated embryonic stem cells lost stemness and deregulated lineage specification. Exposure of trophoblast stem cells to PFI-3 markedly enhanced differentiation [43]. These results emphasize a key function of the BRG1 bromodomain in stem cell maintenance and differentiation. However, PFI-3, unlike BRG1 knockdown, did not change the proliferation rate of triple negative breast cancer cells at any concentration (Figure 2A), nor did it displace BRG1 from chromatin at specific gene loci (Supplemental Figure 4). These results are consistent with a recent study indicating that PFI-3 failed to alter proliferation in multiple tumor cell types in which BRG1 or BRM was mutated or deficient. In that report, the drug was unable to displace endogenous BRM from chromatin, suggesting at least one reason for its lack of effect [44]. The data are also consistent with studies showing that the C-terminal portion of BRG1, which includes the bromodomain, was dispensable for glucocorticoid receptor-mediated gene induction [81] and that the BRG1 ATPase domain, not the bromo domain, is required for leukemia cell proliferation [82].

In contrast, ADAADiN [46, 47] targets the BRG1 ATPase domain and in our study decreased breast cancer cell proliferation (Figure 2B-2E). The specificity of ADAADi for BRG1 in the cells tested was demonstrated by two experiments. First, the combination of ADAADiN treatment and BRG1 knockdown resulted in an inhibition of cell proliferation that was not statistically different from either treatment alone. Second, whereas knockdown of the BRG1 homologue BRM and ADAADiN treatment provided similar inhibition of breast cancer cell proliferation, the combination of the two resulted in an apparent additive effect on proliferation. This suggests that ADAADiN does not or minimally targets BRM in these cells. Thus, one or more molecules that could simultaneously target BRG1 and BRM might have greater potential as a therapeutic agent. Furthermore, ADAADiN treatment, like BRG1 knockdown, increased the sensitivity of triple negative breast cancer cells to chemotherapeutic drugs that are used clinically (Table 1). These results are consistent with the observation that the ATPase domain of BRG1 is required for GR-mediated transactivation [83] and leukemia cell proliferation [82]. In addition, though the chemotherapeutic agents used in this study have diverse mechanisms of action, none directly target an epigenetic regulator, suggesting that combining epigenetic therapies with conventional chemotherapies has great potential in combinatorial drug approaches to treating cancer. Future work will be needed to further explore this possibility. Xenografts and animal models could be used to probe the efficacy of BRG1 inhibitors. Both *in vitro* and *in vivo* approaches to understanding differences in BRG1 functions in normal compared to cancer cells will help inform attempts to improve screening or design of new inhibitors.

The work presented here supports for the idea that targeting BRG1 in breast cancer and in other cancers such as melanoma and colorectal, gastric, and prostate cancer [84–88] could be therapeutic *via* mechanisms that reduce cell proliferation and increase chemosensitivity. Coupled with recent proposals on targeting the BRG1 homologue BRM in BRG1-deficient tumors [89–92], it is apparent that strategies for targeting the human SWI/SNF enzyme ATPases in context-dependent manners will be an expanding area of focus. Indeed, these advances are only part of a larger movement demonstrating that broad classes of epigenetic regulatory proteins are viable targets for novel cancer therapies [93].

### Regulation of ABC transporter genes by BRG1

Reduction of BRG1 levels or interference with BRG1 catalytic function reduces breast cancer cell proliferation, and yet these slow proliferating cells are more sensitive to cytotoxic agents that preferentially target rapidly growing cells. This paradox prompted us to investigate BRG1 effects on the expression of transporters responsible for drug trafficking in cancer cells. Our data show that BRG1 is a regulator of ABC transporters that are implicated as efflux transporters for chemotherapy drugs [94]. ADAADiN inhibited drug-mediated up-regulation of specific transporter genes, indicating a functional role for BRG1. Demonstration that BRG1 was bound to sequences near each transporter gene's transcription start site indicates a direct role for BRG1 during therapeutic drug mediated gene activation. Together these data suggest a possible mechanism for the increased sensitivity of breast cancer cells to chemotherapeutic drugs.

It has been shown that more than half of the ABC transporters are involved in drug resistance using the NCI60 cell line panel [95]. This redundancy in transporter function has limited therapeutic approaches that target specific transporters. For example, MDR1 inhibitors such as zosuquidar and tariquidar failed in clinical trials despite their high potency and specificity [96]. Our discovery that catalytic activity of BRG1 is required for the up-regulation of multiple ABC transporters in response to drug treatment pioneers a new pan-transporter approach to combating drug resistance by targeting BRG1.

## MATERIALS AND METHODS

### Cell culture

MDA-MB-231 cells were obtained from T. Guise [97]. MDA-MB-468 cells were obtained from ATCC. HDQ-P1 cells were purchased from DSMZ (Leibniz Institute DSMZ-German Collection of Microorganisms and Cell Culture, 38124 Braunschweig, Germany. MDA-MB-231, MDA-MB-468 and HDQ-P1 cells were maintained in DMEM supplemented with 10% FBS and Penicillin/Streptomycin. BRG1 knockdown by doxycycline-inducible shRNA expression in MDA-MB-231 cells was performed as previously described [33]. siRNA mediated knockdown of BRG1 in MDA-MB-468 and HDQ-P1 cells was performed using reagents and methods previously described [33, 98]. The identities of all four triple negative breast tumor lines were authenticated by Short Tandem Repeat profiling at the Genetic Resources Core Facility, Johns Hopkins School of Medicine, Institute of Genetic Medicine.

### Reagents

5-FU, cyclophosphamide, gemcitabine, doxorubicin, cisplatin, paclitaxel, and 3-(4,5-dimethyl-2-thiazolyl)-2, 5-diphenyl-2H-tetrazolium bromide (MTT) were purchased from Sigma-Aldrich (St. Louis, MO). ^3^H-Paclitaxel and ^14^C-5-Fluorouracil were purchased from Moravek Biochemicals (Brea, CA). PFI-3 was purchased from Xcess Biosciences, Inc (San Diego, CA). ADAADiNN was prepared from neomycin as previously described [46]. CellTiter 96 AQueous One Solution was purchased from Promega (Madison, WI).

### Drug treatment

Cells were plated and incubated overnight before treatment with increasing doses of drugs for 72 hours to determine the IC50 value. When combined with ADAADiN treatment, cells were pre-treated with 2 μM ADAADiN for 48 hours and then different drugs were added to culture medium at the IC50 value incubated for another 24 hours and collected for analysis.

### Drug uptake and retention studies

MDA-MB-231 scram and shBRG1 cells were treated with doxycycline to induce BRG1 knockdown as described previously [33]. Cells were then treated with 0.1 μCi ^3^H-Paclitaxel or ^14^C-5-Fluorouracil for 1 hour or 6 hours, respectively. Uptake of radiolabeled drug was measured after washing the cells repeatedly, cell counting, and scintillation counting. For assessing drug retention, labeled cells were washed 3 times with PBS to remove residual labeling medium and replaced with growth medium containing doxycycline and 100 μM paclitaxel or 1 mM 5-FU for an additional 2 hours before harvest. All cells, including any floating cells, were collected, counted and lysed by addition of 0.5 N NaOH. Cell lysates were analyzed by scintillation counting. Readouts were normalized by cell number.

### MTS assay

Cells were seeded in 96-well plates (5,000 cells/well) overnight prior to drug treatment, and were then maintained in the presence of vehicle or drug for 72 hours before addition of 20 μL CellTiter 96 AQueous One Solution per well. Plates were incubated for 2 hours before absorbances at 490 nm were measured with a Synergy H4 Hybrid microplate reader (Bio Tek, Winooski, VT).

### MTT assay

Cells were seeded in 96-well plates (5,000 cells/well) overnight prior to drug treatment, and were then maintained in presence of vehicle or drug for 72 hours before addition of MTT solution (5 ug/mL). Plates were incubated for 4 hours in MTT solution, then the media was removed and plates were air-dried. One hundred microliters of DMSO were added to each well and incubated at room temperature for 30 minutes with gentle shaking before the absorbances were measured as described above.

### Chromatin immunoprecipitation (ChIP)

ChIP was performed as described previously [99] with the following modifications: cells were cooled to room temperature before being cross-linked with ice-cold growth medium containing 3.7% formaldehyde for 40 min at 4°C. 50 μg of chromatin extract was used for each ChIP with 10 μL of BRG1 antisera or normal rabbit IgG (Millipore, Billerica, MA). BRG1 binding at sequences at transporter genes was measured by real time qPCR using primers listed in Supplemental Table 1.

### RT-qPCR

Total RNA was extracted from one million cells using RNeasy Plus following manufacturer's instruction (Qiagen Inc., Valencia, CA). cDNA was synthesized using SuperScript III kit (Invitrogen, San Diego, CA). Gene expression was measured by real time qPCR on a StepOne Plus realtime PCR System (Applied BioSystems, Grand Island, NY) using the primers listed in Supplemental Table 2. Relative gene expression was normalized to beta actin in each sample in experiments comparing vehicle *vs* drug treatment. In experiments comparing scram and shBRG1 samples, expression was calculated relative to 45S pre-rRNA.

### Survival analysis

Kaplan-Meier survival curves were plotted using GraphPad Prism 5.0 software and statistical significance was analyzed using the log-rank test.

### Statistical analyses

All quantitative data points represent the mean of three independent experiments performed in duplicates or triplicates with standard deviation (S.D). Unless indicated, statistical analysis was performed using GraphPad Instat two-tail *P* value student test (Graphpad Software, Inc., La Jolla, CA). The significance of the correlation between BRG1 expression and the expression of each of the transporter genes was determined by calculating the Pearson coefficient. The lines present in the graphs shown in Supplemental Figure 5 were determined by linear regression.

## SUPPLEMENTARY MATERIALS FIGURES AND TABLES


