# Supplementary material for: Targeting the chromatin remodeling enzyme BRG1 increases the efficacy of chemotherapy drugs in breast cancer cells
Source: Oncotarget. 2016 Mar 25;7(19):27158–75. doi: 10.18632/oncotarget.8384 (PMC5053639; doi:10.18632/oncotarget.8384)
Supplement: Supplementary file 1 [file oncotarget-07-27158-s001.pdf]

Targeting the chromatin remodeling enzyme BRG1 increases the efficacy of chemotherapy drugs in breast cancer cells

Supplemental Material

Supplemental Figure 1

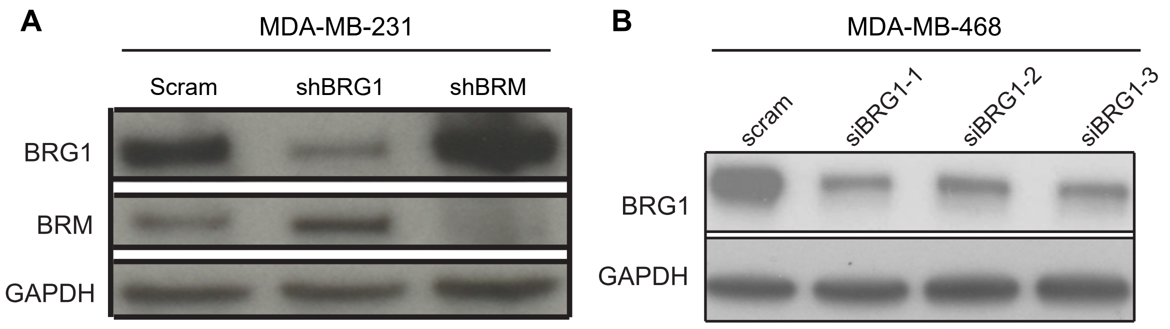

Supplemental Figure 1: Western blot validation of knockdown in MDA-MB-231 and MDA-MB-468 cells. (A) BRG1 or BRM was knocked down in MDA-MB-231 cells 72 hours after doxycycline induction of shRNA. Cells expressing a scrambled sequence shRNA (scram) were used as a control. The increase in BRM protein levels in BRG1 knockdown cells and the increase in BRG1 protein levels in BRM knockdown cells were previously described [1]. **(B)** BRG1 knockdown in MDA-MB-468 cells by three different siRNAs compared to a scrambled sequence siRNA (scram). GAPDH levels were monitored as a loading control.

Supplemental Figure 2

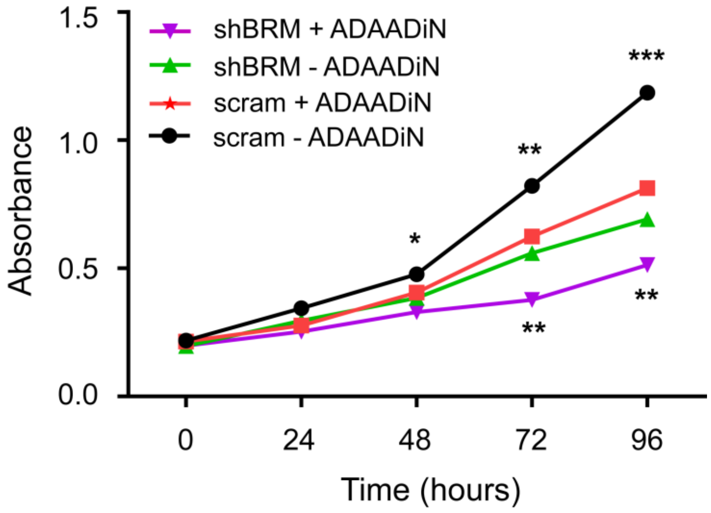

Supplemental Figure 2: Effects of BRM knockdown on ADAADiN inhibition of cell proliferation. Cell proliferation was measured by MTT assay after ADAADi treatment, inducible BRG1 knockdown, or both in MDA-MB-231 cells. Each data point is the mean from 3 independent experiments performed in triplicate; error bars are standard deviations. *P<0.05, **P<0.01, ***P<0.001.

Supplemental Figure 3

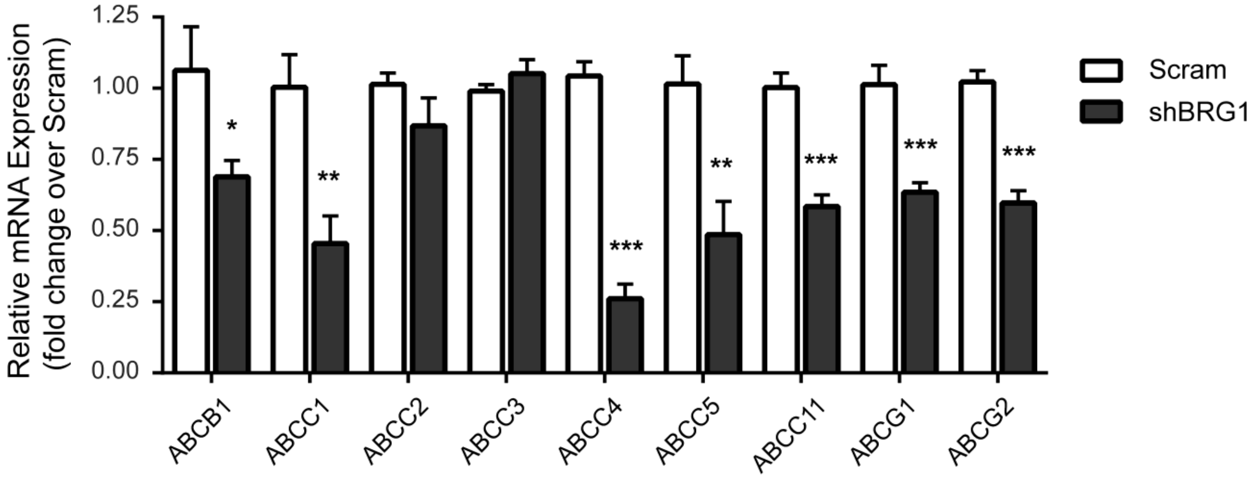

Supplemental Figure 3: BRG1-dependence of endogenous ABC transporter gene expression. Bar graphs present relative mRNA expression of specific ABC transporter genes relative to the expression of 45S pre-rRNA in MDA-MB-231 cells expressing a control shRNA (scram) or shRNA targeting BRG1. Data are expressed as fold-change relative to scram cell values. Each bar presents the mean of 3 independent experiments performed in duplicates; error bars are standard deviations. *P<0.05, **P<0.01, ***P<0.001.

Supplemental Figure 4

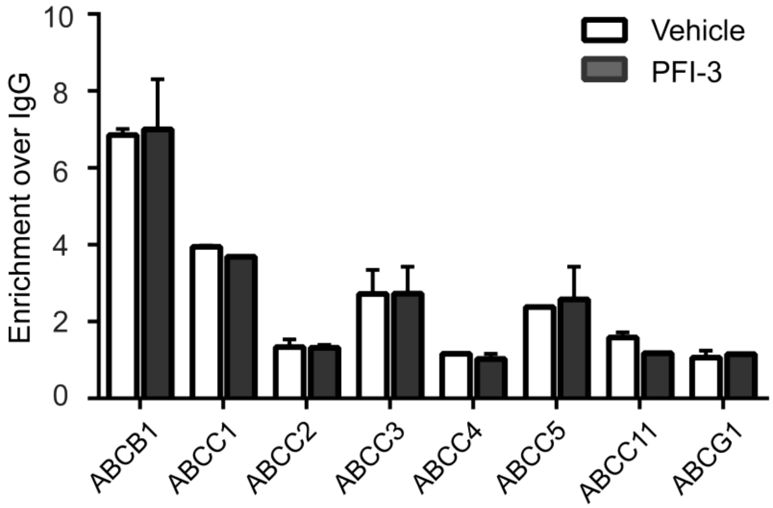

Supplemental Figure 4: BRG1 binding at ABC transporter gene loci in the presence or absence of PFI-3. ChIP was performed using MDA-MB-231 cells that were treated with vehicle or PFI-3. BRG1 binding at transporter genes was measured by quantitative PCR using primers listed in Supp. Table 2. The bars represent the ratio (enrichment) of BRG1 binding to sequences near the indicated transporter gene relative to signal obtained by pulldown with IgG instead of the antibody against BRG1. Each bar presents the mean of 3 independent experiments performed in triplicate; error bars are standard deviations. *P<0.05, **P<0.01, ***P<0.001.

Supplemental Figure 5

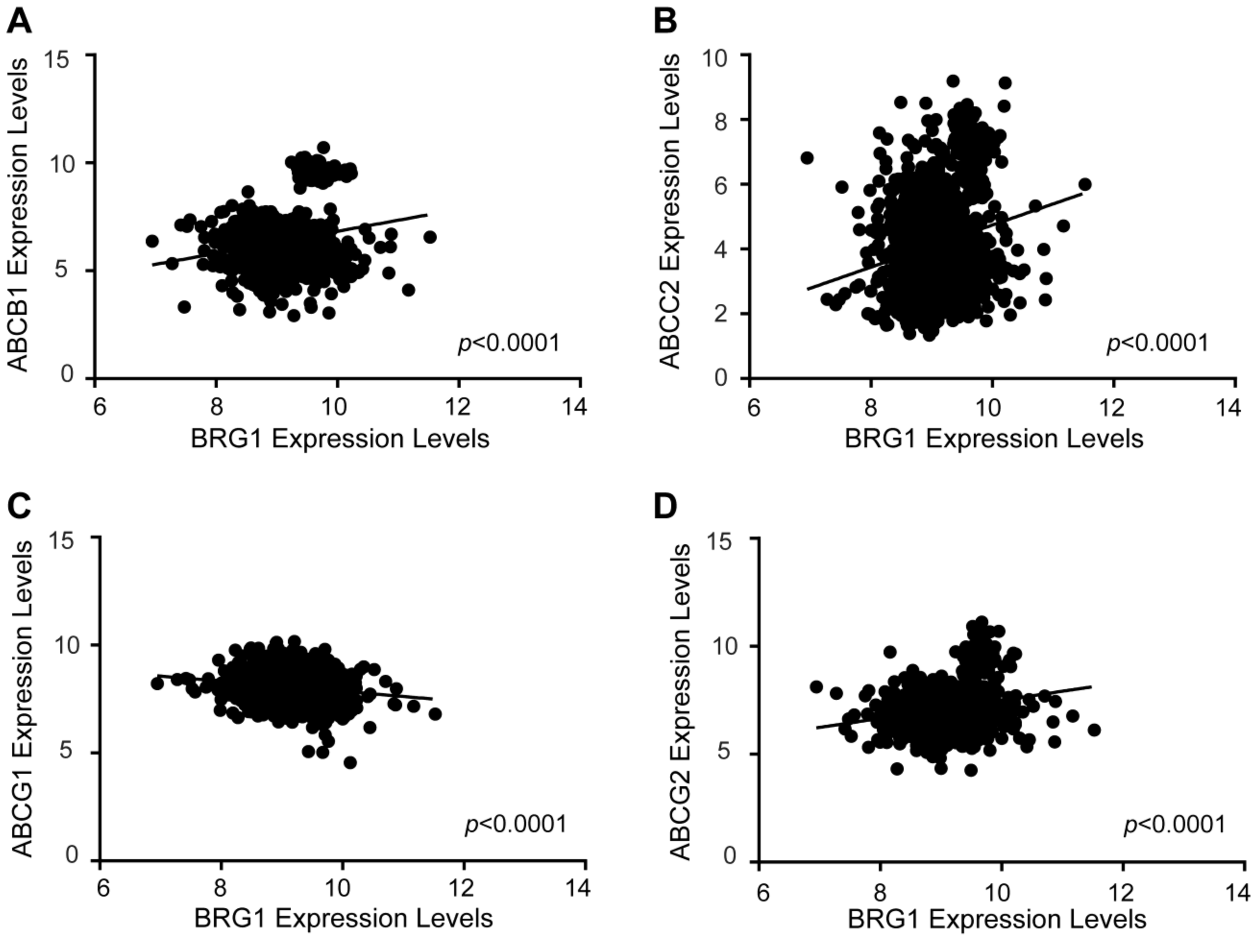

Supplemental Figure 5: Relationship between ABC transporter and BRG1 expression in patient tumors. Expression levels of **(A)** ABCB1 **(B)** ABCC2 **(C)** ABCG2 **(D)** ABCG1 were extracted from expression data from human breast cancer patients in 7 combined datasets and were plotted against BRG1 expression from the same patients. Values on the x- and y-axes are the log2 value of normalized expression levels for the indicated gene. The significance of the correlation between BRG1 and each of the transporter gene expression was determined by calculating the Pearson coefficient. The line shown in each graph was determined by linear regression.

Supplemental Table 1: ChIP Primers

Gene	Forward Primer	Reverse Primer
ABCB1	TAAGGCAGGCAGGCTTGAAA	CTACTCCCACCCTTCCTCCA
ABCC1	GCCTGTAATCCCAGCCCTTT	ACAGGGTTTCACCAGGTTGG
ABCC2	AAATGCTCTGAGCTCCACCT	CAGAGGTTGCCAGGGGTTAG
ABCC3	ATTCAGGAGGGAGCTTTGCC	CCATTTCCCTGTCTGGGGAC
ABCC4	TTCTCAGGACCAAACGACGG	CCCGGCTTTCTTGAGGTCTT
ABCC5	CAGTGCTGTTCGTAGGCAGA	AGGGTCGAACCATGAGCAAG
ABCC11	GCCCTTTACTCCTATCCGCC	AGAATGCCTCAGCGGACAAA
ABCG1	CCTACTGAGGAGGGCATGGA	TGTCTTGTTCCAGCTGTGGC

Supplemental Table 2: RT-qPCR Primers

Gene	Forward Primer	Reverse Primer
ABCB1	CGTGGTTGGAAGCTAACCCT	CAGCAGCTGACAGTCCAAGA
ABCC1	TCACCATGCTGCTTGCTACC	ATCTCAGGATGGCTAGGGCA
ABCC2	TCCCTGTCCCTAGGGCTTTT	AGGATGACCTTTCATCCCAACC
ABCC3	AAAAGCAGACGGCACGACAC	AGGCATTTTTCCCAGGTGCT
ABCC4	TCTGCTCACGCGTGTTCTTC	ATCCCAGAACCCTTGCAACTC
ABCC5	CGGCCGATGCCGCTATAAA	CATCAGAATTCCTGCGCCCC
ABCC11	ACCAGGTTGGCAAATCATACT	ATCGCCTATGTCGATGCCA
ABCG1	GCTTTCTCGGTCGGCACC	ACCTCATCCACCGAGACACA
ACTB	CATGTACGTTGCTATCCAGGC	CTCCTTAATGTCACGCACGAT
45S pre-rRNA	CTCCGTTATGGTAGCGCTGC	GCGGAACCCTCGCTTCTC

References

1. Wu Q, Madany P, Akech J, Dobson JR, Douthwright S, Browne G, Colby JL, Winter GE, Bradner JE, Pratap J, Sluder G, Bhargava R, Chiosea SI, van Wijnen AJ, Stein JL, Stein GS, et al. The SWI/SNF ATPases Are Required for Triple Negative Breast Cancer Cell Proliferation. J Cell Physiol. 2015; 230(11):2683-2694.
